# Research progress in the preparation of sodium-ion battery anode materials using ball milling

**DOI:** 10.1039/d4ra08061k

**Published:** 2025-02-25

**Authors:** Liwen Zhang, Shandong Huang, Yihong Ding, Tianbiao Zeng

**Affiliations:** a Key Laboratory of Carbon Materials of Zhejiang Province, Wenzhou Key Lab of Advanced Energy Storage and Conversion, Zhejiang Province Key Lab of Leather Engineering, College of Chemistry and Materials Engineering, Wenzhou University Wenzhou 325035 China 20195205@wzu.edu.cn; b Research Institute of Interdisciplinary Sciences (RISE) and School of Materials Science & Engineering, Dongguan University of Technology Dongguan 523808 China tianbiaozeng@163.com

## Abstract

Sodium-ion batteries are regarded as one of the most promising alternatives to lithium-ion batteries due to the greater abundance and lower cost of sodium compared to lithium. However, sodium-ion batteries have not yet been widely adopted. The main reason is that, compared to lithium-ion batteries, sodium-ion batteries have lower energy density and shorter cycle life, with the performance of anode materials directly affecting the energy density and cycle stability of sodium-ion batteries. Notably, ball milling, as an efficient material processing technique, has been widely applied in the preparation and modification of sodium-ion battery anode materials in recent years. This paper reviews the recent progress in the preparation of sodium-ion battery anode materials using ball milling. The process is categorized into ball milling mixing, ball milling exfoliation, ball milling synthesis, and ball milling doping. First, the basic principles and mechanisms of ball milling technology are introduced. Then, the preparation of different types of sodium-ion battery anode materials is discussed based on four specific categories. For various material systems, the effects of ball milling on the structure, morphology, and electrochemical performance are discussed. Additionally, the advantages and challenges of using ball milling in the preparation of sodium-ion battery anode materials are summarized. Finally, the future directions and development trends in the preparation of sodium-ion battery anode materials using ball milling are forecasted, aiming to provide insights and references for further research in this field.

## Abstract 2

Ball milling, as an efficient material processing technique, has been widely applied in the preparation and modification of sodium-ion battery anode materials in recent years.

## Graphical abstract

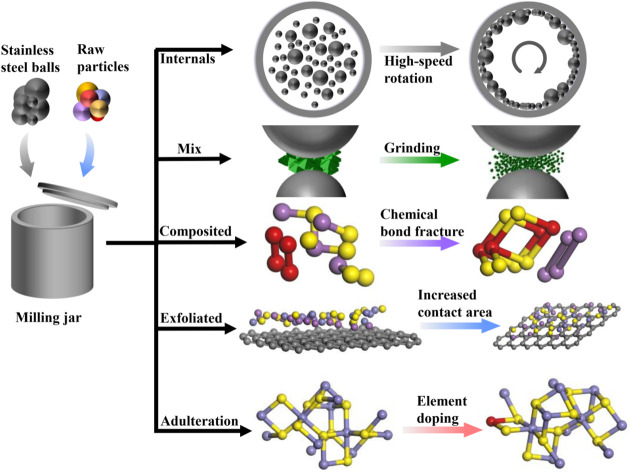

## 1. Introduction

With the continuous development of the economy, energy consumption and resource shortages have posed significant threats to both the natural environment and human development. As a result, the search for renewable energy sources has gradually become a pressing issue, attracting widespread attention from scholars.^1,2^ Although renewable energy sources (such as wind, hydro, solar, and tidal energy) can reduce environmental pollution and resource waste, their production is often unstable due to the influence of weather and natural conditions. Additionally, the relatively immature technology for energy storage and transmission can lead to further waste and losses.^3^ Therefore, devices capable of reliably storing and releasing energy are urgently needed.^4^ At the same time, new energy storage technologies have gradually gained recognition, becoming a current research hotspot.^5^ Common new energy storage devices include hydrogen storage systems,^6,7^ supercapacitor storage systems,^8^ lithium-ion battery storage systems,^9,10^ and solar cell storage systems.^11^ Among them, lithium-ion batteries are particularly favored for their long cycle life, high energy density, and wide operating temperature range. However, due to their high cost, scholars have been seeking alternative energy storage systems.^12^ Sodium, being in the same group as lithium on the periodic table, offers a much lower cost for battery-grade sodium salts—less than 1/30 of lithium salts. Additionally, sodium-ion batteries share similar electrochemical mechanisms with lithium-ion batteries. Moreover, sodium-ion batteries possess advantages such as thermal stability and excellent deep charge/discharge performance,^13^ positioning them as a potential alternative to lithium-ion batteries for energy storage. The electrode materials in sodium-ion batteries include cathode materials (polyanionic compounds,^14,15^ Prussian blue analogs,^16,17^ and layered oxides^18^) and anode materials (carbon materials,^19^ titanium-based materials,^20^ organic materials,^21^ and alloy materials^22,23^). Compared to cathode materials, the instability and lower energy density of anode materials limit the performance of sodium-ion batteries.^24^ Therefore, improving anode materials is a key research direction in the study of sodium-ion batteries. Many scholars are dedicated to exploring new preparation techniques to enhance the electrochemical performance of anode materials.

In recent years, ball milling technology has become a highly popular material preparation technique due to its ability to refine grains, enhance powder activity, and improve particle distribution uniformity. In the preparation of sodium-ion battery anode materials, researchers typically focus on sodium-ion insertion materials, conversion reaction materials, alloying reaction materials, and organic anode materials, depending on the substances and objectives involved in the ball milling process. During the ball milling process, they may mix different raw materials to achieve the desired chemical composition, synthesize materials with specific structures, dope external atoms or molecules to enhance electrochemical performance, and exfoliate or regulate the surface morphology of the materials, ultimately improving battery cycle life and performance stability.

This paper reviews the latest progress in the preparation of sodium-ion battery anode materials using ball milling technology, covering the following aspects. (1) The process and application of ball milling technology in the preparation of sodium-ion battery anode materials are introduced. (2) The review categorizes and presents the ball-milled carbon-based materials (graphite, carbon nanotubes, graphene, *etc.*), including milling of single substances to alter their crystal structure, milling of mixed carbon-based materials, milling with phosphorus, and milling with sulfides, selenides, and oxides. (3) It describes the use of ball milling for exfoliation to synthesize materials, primarily exfoliating graphite to mix with other active materials (FeSb_2_S_4_, Sb/Fe_2_S_3_, and SeP_2_). (4) The paper details the synthesis of new composite anode materials through ball milling, such as the separate or combined preparation of phosphorus and carbon-based materials with metals, non-metals, oxides, sulfides, fluorides, and polymers. (5) It provides a detailed discussion based on the types of dopants used during ball milling, including elemental doping, ionic doping, and functional doping, and discusses the advantages of the ball milling doping method compared to other doping techniques. (6) Finally, the review summarizes the above findings and discusses the challenges and feasibility of further developing high-performance sodium-ion battery anode materials.

## 2. Ball milling technology

**Fig. 1:**
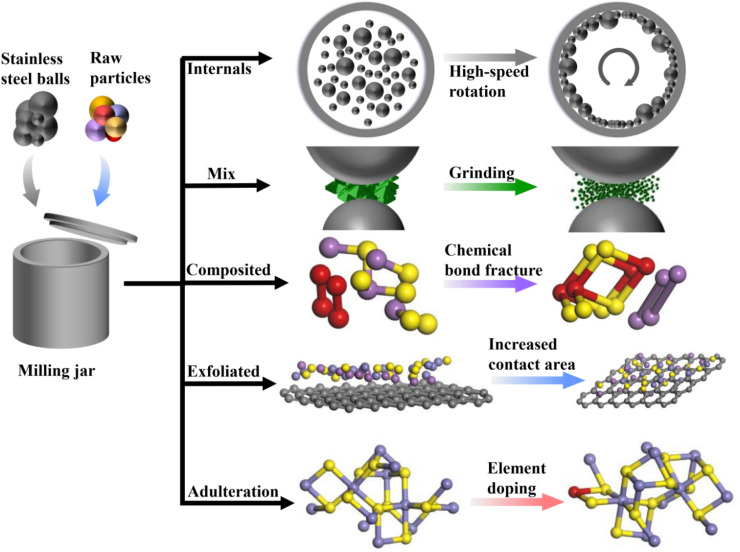
Working principle of the ball milling jar and potential changes in the raw material particles inside.

Ball milling technology, as an energy-saving and efficient material preparation technique, has been widely applied in the research of sodium-ion battery anode materials. This technique involves the high-speed collision of stainless steel or zirconia balls in the milling jar, ensuring that the materials inside are thoroughly ground. Notably, during the ball milling process, the raw material particles are subjected to continuous high-energy impacts, compression, and friction from the balls, as shown in Fig. 1, which may alter the structure of the material or further promote chemical reactions between different substances.

First, the mechanical activation effect occurring during the ball milling process can significantly alter the microstructure of the raw material particles. The raw material particles undergo repeated collisions and grinding, resulting in a gradual reduction in size and an increase in surface area, which leads to significant changes in the microstructure of the material particles. For example, Pradeep *et al.* conducted X-ray diffraction (XRD) tests on mixtures of Ti, Mg, and Sr elements under different ball milling durations. They observed that as the milling time increased, the powder particles underwent continuous deformation, leading to a phase transition from crystalline to amorphous. This process effectively altered the microstructure of the mixture.^25^ Additionally, mechanical activation may also lead to changes in the internal structure of the raw material particles, such as distortion of the crystal structure, bond breakage, and atomic rearrangement, which in turn affect the electrochemical performance of the materials. Secondly, chemical reactions may be triggered during the ball milling process. Under the influence of high-energy balls, the raw material particles may undergo redox reactions, syntheses, and other chemical changes. These chemical reactions often facilitate the formation of new phases or alter the composition and structure of existing materials, thereby affecting their electrochemical performance. For instance, Tan *et al.* demonstrated a simple ball milling process for the preparation of CuS@rGO composites, which effectively proved through comparative experiments that a redox reaction occurred during ball milling, leading to the formation of rGO. In Tan's work, a water-based copper–aluminum mixed ion battery was prepared using CuS@rGO as the anode material, which retained 80% of its initial capacity after 500 charge–discharge cycles at a rate of 5 A g^−1^, providing an effective strategy for developing electrode materials for copper–aluminum mixed ion batteries with high current stability.^26^ Furthermore, local thermal effects may also accompany the ball milling process. Inside the rapidly rotating ball milling jar, the raw material particles are subjected to impacts and friction from high-energy balls, generating local high temperatures, which may trigger pyrolysis, thermal decomposition, and other thermal effects, further affecting the structure and performance of the materials. Piras *et al.* reviewed the potential of ball milling technology in the field of cellulose nanoparticles, particularly in the preparation and chemical modification of cellulose nanocrystals and nanofibers. During the separation of cellulose nanoparticles, ball milling technology may be influenced by thermal effects, making the optimization of this method particularly important.^27^

Of course, milling speed, milling time, and the ball-to-powder ratio are key parameters in ball milling technology. Typically, optimal milling parameters result in appropriate energy input, which breaks down material agglomeration and forms a uniform distribution of the material. For example, Li *et al.* used wet ball milling to prepare graphene nanosheets (GNSs)/Al_2_O_3_ composites and systematically studied the effect of milling speed on the layered structure, dispersion, and conversion efficiency of graphene in the composites.^28^ The study showed that as the milling speed increased within the range of 200–300 rpm, the conversion efficiency of graphene significantly improved. Specifically, at 300 rpm, the conversion efficiency of graphene reached its maximum, while the number of graphene layers gradually decreased to below 10, showing better dispersion and optimization of the interlayer structure. During this process, the intensity of the graphite peak of graphene gradually weakened, indicating that milling accelerated the interlayer slip and reconstruction of graphene, which in turn improved its dispersion. Notably, no characteristic peaks of graphene oxide (GO) appeared in the composites at different milling speeds, suggesting a low oxidation degree of graphene and effectively avoiding the detrimental effects of excessive oxidation on performance. Meanwhile, as the milling speed increased, the interaction between graphene and Al_2_O_3_ nanoparticles strengthened, resulting in a more uniform and stable composite structure. These results suggest that milling speed is a critical factor influencing material performance. Properly controlling the milling speed helps improve the dispersion of graphene in the composites, increase its conversion efficiency, and optimize the electrochemical performance of the composites. Additionally, Wang *et al.* studied the effect of different milling times on the dispersion behavior and microstructural evolution of graphene nanoplates (GNPs) and TiB whiskers (TiBw) particles in a Ti–6Al–4V (TC4) matrix.^29^ The study showed that as the milling time increased, the GNPs in the TC4 matrix gradually transitioned from an agglomerated state to a more uniform dispersion. When the milling time was 2 and 4 hours, GNPs failed to disperse effectively, exhibiting significant agglomeration and resulting in uneven distribution. The GNPs had a higher number of layers, with a loosely covered surface. As the milling time was extended to 8 hours, most of the GNPs were evenly distributed on the surface of the TC4 powder, maintaining their original wide sheet structure and exhibiting improved mechanical properties. After increasing the milling time to 16 and 24 hours, the dispersion of GNPs further improved. However, excessive milling damaged the structural integrity of the graphene, resulting in an increase in defects and a decrease in the number of graphene layers. These results indicate that an appropriate milling time is crucial for maintaining the structural integrity and uniform dispersion of GNPs, which is key to achieving excellent performance of GNPs in TC4-based composites. Excessive milling time, however, can negatively affect the structure of graphene. Ning and his colleagues found that the ball-to-powder ratio significantly affected the morphology and structure of carbon fiber materials when using high-energy ball milling to prepare an oxygen-doped carbon material.^30^ Specifically, at a ball-to-powder ratio of 20 : 1, the milled material exhibited an amorphous-like structure, with shorter carbon layers, indicating a low degree of graphitization and a high number of defects. When the ball-to-powder ratio increased to 80 : 1, ordered graphite layers were unexpectedly observed at the carbon edges. These graphite domains were very thin, only a few nanometers thick, while the internal carbon layers remained highly disordered. As the ball-to-powder ratio was further increased to 200 : 1, the carbon fibers were completely pulverized into small particles, and the graphitization degree of the carbon layers significantly improved. The carbon layers at the edges became more ordered, forming distinct graphite layer structures. As the ball-to-powder ratio increased, the degree of graphitization and the structural order of the material gradually improved, and the particle size became more uniform, thereby significantly enhancing the electrochemical performance of the material. These results suggest that the ball-to-powder ratio is a key factor influencing the morphology and graphitization process of carbon fibers. Proper control of the ball-to-powder ratio not only optimizes the morphology of carbon fibers but also significantly enhances their structural order and conductivity, thereby improving the final material's performance. Therefore, controlling the appropriate milling parameters has a significant impact on material performance. Properly adjusting these parameters can not only improve the dispersion of the material but also optimize the microstructure and overall performance of the composite material.

Compared to other common preparation methods for sodium-ion battery anode materials, ball milling offers significant advantages, including simplicity, low cost, and high raw material utilization, making it highly suitable for large-scale production. Thanks to intense mechanical forces, ball milling can efficiently achieve uniform mixing and doping of multi-component materials, significantly enhancing the conductivity and electrochemical performance of the materials, particularly showing excellent results in the preparation of composite and doped materials. Furthermore, ball milling does not rely on complex solvents or expensive equipment, offering excellent industrial production potential and providing a feasible technological pathway for large-scale production. However, a disadvantage of ball milling is its poor control over the material morphology, which may have some impact on the final material's performance. Table 1 summarizes and compares the main methods used in recent years for the preparation of sodium-ion battery anode materials.

**Table 1:** Main methods for preparing sodium-ion battery anode materials in recent years

Material preparation methods	Composite	Morphological features	Specific surface area (m^2^ g^−1^)	Current density	Capacity retention (mA h g^−1^)	Advantages	Disadvantages	Ref.
Ball milling method	Red-P/BaTiO_3_/graphene	Uniformly distributed nanosheet structure	NA	200 mA g^−1^	823 (100 cycles)	High raw material utilization; simple operation; low cost; good uniformity; environmentally friendly	Material morphology is less controllable	31
	Amorphous P_4_SSe_2_	Irregularly rough particulate structure	NA	100 mA g^−1^	937 (80 cycles)			32
	Na_2_S_*x*_-hard carbon-2	Surface-damaged monodisperse spheres	10.68	2000 mA g^−1^	250.8 (3000 cycles)			33
	Oxygen-rich carbon	Irregular particulate form	72.9	1000 mA g^−1^	250 (1000 cycles)			30
	SeP@high-conductivity crystalline graphene	Nanoparticulate form	NA	650 mA g^−1^	732 (500 cycles)			34
Sol–gel method	Co_9_S_8_@NPC	Porous 3D network structure	68.2	500 mA g^−1^	120.4 (3000 cycles)	Materials with special structures and morphologies can be obtained	Complex process; low yield; potential pollution	35
	Na_0.66_Li_0.22_Ti_0.78_O_2_	Smooth-surfaced particulate form	2.1	1C	104 (250 cycles)			36
	15 wt% V^5+^-doped Na_2_Ti_3_O_7_	Nanorod structure	NA	100 mA g^−1^	136 (900 cycles)			37
	NaV_2.91_Mn_0.09_(PO_4_)_3_/C	Aggregated nanorod structure	NA	10C	121.1 (5000 cycles)			38
Hydrothermal method	BiVO_4_–V_2_C	Embroidered spherical heterostructure	32.03	1000 mA g^−1^	303 (2000 cycles)	Controllable particle size and morphology; mild reaction conditions; high purity and high crystallinity	High temperature and pressure requirements; limited solvent selection	39
	Sb_2_Se_3_@g-C_3_N_4_	Rod-like structure with a rough surface	NA	5000 mA g^−1^	275 (600 cycles)			40
	TiO_2_ micro/nano-spheres	Urchin-like micro/nanosphere structure	210.7	1000 mA g^−1^	204.5 (1000 cycles)			41
	M-FeS@C	Sheet-like structure	2.3	1000 mA g^−1^	372 (500 cycles)			42
	5% Co–Fe_3_O_4_/rGO	Oval-shaped nanoparticle structure	NA	0.05C	364.6 (100 cycles)			43
Electrodeposition method	Sb@3D-Cu	Pine-needle-like Sb clusters adhered to the 3D-Cu substrate	NA	1000 mA g^−1^	289.6 (200 cycles)	Strong controllability; fast electroplating speed and low cost	Only applicable to conductive substrates; limited deposition layer thickness	44
	Sb@In_2_O_3_-100 s	3D porous structure	NA	300 mA g^−1^	456.5 (300 cycles)			45
	Sb–Zn alloys	Agglomerate structure with appropriate porosity	NA	300 mA g^−1^	266 (320 cycles)			46
	Sb_47_Fe_39_P_14_	Ring-shaped particulate structure	NA	100 mA g^−1^	431.4 (200 cycles)			47
CVD	Co-, N-modified porous carbon fibers@SnS@G	Fibrous structure	101.7	2000 mA g^−1^	541.4 (100 cycles)	Strong controllability; wide applicability	High temperature and complex equipment requirements; harmful gases, and solvent contamination issues	48
	Carbon microspheres self-supported on 3D Ni foam	Onion-like structure	63	20 mA g^−1^	136.2 (750 cycles)			49
	NG@SnSe/C	Nanosheet structure	NA	500 mA g^−1^	405.4 (100 cycles)			50
	TiC nanoflowers	Porous nanoflower-like structure	NA	1000 mA g^−1^	73.5 (2500 cycles)			51
Electrospinning method	C@SbPO_4_	Porous fibrous sheet-like structure	35.45	100 mA g^−1^	363 (300 cycles)	High specific surface area; easy to operate	Post-processing required; low yield; potential pollution	52
	CoFe_2_O_4_@carbon@alkalized MXene	Nanofibrous structure	37.2	100 mA g^−1^	416 (500 cycles)			53
	Co_9_S_8_/WS_2_@C@CNF	Carbon nanofibrous structure	379	2000 mA g^−1^	378.8 (700 cycles)			54
	CoTe_2_/Sb_2_Te_3_@NCNFs	Porous network structure	159.48	200 mA g^−1^	301.77 (500 cycles)			55
	ZnSe/SnSe@Se,N-CNFs	Nanofibrous structure	NA	2000 mA g^−1^	587.5 (3500 cycles)			56

## 3. Application of ball milling technology in the preparation of sodium-ion battery anode materials

### 3.1 Pulverization/mixing of sodium-ion battery anode materials

**Fig. 2:**
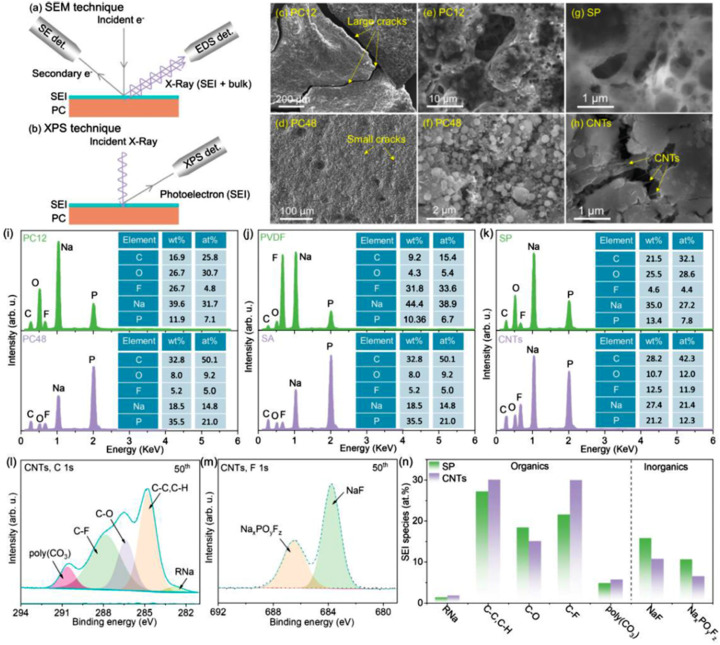
Optimization mechanism for advanced PC electrodes (a) and (b) operation principle schematics of SEM (a) and XPS (b) technique. SE det. stands for secondary electron detector (c) and (d) low magnification SEM images of PC12 electrode (c) and PC48 (d) electrode after 3 cycles (e) and (f) high magnification SEM images of PC12 electrode (e) and PC48 electrode (f) after 3 cycles (g) and (h) SEM images of PC48 electrode with PVDF (g) and SA (h) binders after 3 cycles. (i) EDS profiles and corresponding element ratio of PC12 and PC48 electrode after 3 cycles (j) EDS profiles and corresponding element ratio PC48 electrode with PVDF and SA binders after 3 cycles. (k) EDS profiles and corresponding element ratio PC48 electrode with SP and CNT-conductive additives after 50 cycles (l)–(n) C1s (l) and F1s (m) XPS of PC48 electrode with CNTs conductive additives after 50 cycles, and corresponding SEI species ratio (n) of PC48 electrode with SP and CNTs conductive additives.^60^

Sodium-ion batteries have tremendous potential as a new generation of energy storage devices, particularly in the context of low costs and abundant resources, making them a focus of attention. However, compared to lithium-ion batteries, sodium-ion batteries face different challenges in the selection of anode materials. Hard carbon is a typical anode material for sodium-ion batteries, characterized by its disordered, non-layered structure, which allow reversible storage of sodium ions through sodium adsorption. To enhance the electrochemical performance of hard carbon materials in batteries, the particle size of the materials is crucial. Typically, hard carbon materials exist in bulk form and need to be converted into uniform granules through a pulverization process to provide a larger specific surface area and more effective sodium ion diffusion pathways.^57^ Ilic *et al.* utilized a planetary ball mill to mill hard carbon and compared it with unmilled hard carbon, elucidating the changes in porosity during the milling process and confirming that materials with larger closed pore volumes store more charge at low pressure.^58^ Additionally, Bommier *et al.* achieved a reversible capacity of 335 mA h g^−1^ at a current of 40 mA g^−1^ using hard carbon with measurable low specific surface area/porosity, as well as 500 cycles at 300 mA g^−1^.^59^ However, Clement also indicated the need to seek lower porosity to further enhance energy density while reducing irreversible capacity. Therefore, it is crucial to design an anode material that can achieve both high reversible capacity and long-term cycling stability. Mixing hard carbon with other materials is an effective strategy to enhance electrode performance, as hard carbon possesses good conductivity and structural stability, which can maintain the overall morphology of the material during charge and discharge cycles, thereby reducing the risk of structural collapse. However, the capacity of hard carbon is relatively low; if it can be combined with other active materials, the synergistic effect between the materials can not only enhance sodium ion diffusion pathways but also improve overall capacity and cycling stability. For example, Zhou's research group conducted a mixed ball milling treatment of phosphorus and carbon to prepare phosphorus/carbon (PC) composite materials, optimizing the PC composites by adjusting ball milling time, binders, and conductive and electrolyte additives, their work is shown in Fig. 2.^60^ The research results indicate that extending the ball milling time helps reduce particle size, thereby alleviating the volumetric expansion of the material. Furthermore, hybridizing hard carbon (HC), which has a low potential, good stability, and high conductivity, with PC, which offers high capacity, high initial efficiency, and reasonable rate performance, is the preferred strategy for constructing sodium-ion battery anodes. The energy density has increased to 150 W h kg^−1^; however, various deficiencies remain, including the need to improve coulombic efficiency and energy efficiency.

At this point, the use of bamboo-derived hard carbon (BB) to prepare carbon-based anode materials attracted the attention of Pothaya and colleagues.^61^ Bamboo, as a natural porous material, is not only environmentally friendly but also reduces the cost of active materials; its unique three-dimensional open framework structure allows for rapid movement of sodium ions and electrons, thereby shortening the diffusion path of sodium ions. Pothaya found that the specific discharge capacity of bamboo-derived hard carbon as an anode for sodium-ion batteries was 197.5, 167.3, 108.45, and 47.2 mA h g^−1^ at 0.1C, 0.2C, 1.0C, and 5.0C, respectively. It can be seen that its electrochemical performance still needs improvement, leading to the choice of combining it with carbon nanotubes (CNT). CNT, which are tubular structures formed by carbon atoms arranged in a hexagonal pattern, serve as a support and conductivity enhancer for sodium-ion battery anode materials, significantly promoting the electrochemical performance and cycling life of the batteries. Based on this, the authors synthesized a composition of CNT and BB using a ball mill, with CNT contents set at 1 wt%, 5 wt%, and 10 wt%, respectively. At 5 wt%, the sample exhibited excellent performance and outstanding stability. The initial discharge capacity was 268.9 mA h g^−1^ at 0.1C, and the ball milling process greatly facilitated the incorporation of carbon nanotubes into hard carbon, inducing the formation of nanopores and enhancing the rate performance of the sodium-ion battery. Therefore, other carbon-based materials (such as carbon black and carbon nanotubes) have also attracted attention.

Both carbon black and carbon nanotubes have high specific surface areas, allowing them to provide more active sites, thereby enhancing the rate and efficiency of electrochemical reactions. Furthermore, due to their structural characteristics, carbon black and carbon nanotubes possess high flexibility, allowing them to adapt well to volume changes during battery charging and discharging processes, thus extending the battery's cycle life. For instance, Güneren *et al.* used carbon black in conjunction with amorphous SiOC powder synthesized through thermal crosslinking and distributed pyrolysis, grinding them under low-energy and high-energy milling conditions (LEM and HEM), they found that the SiOC obtained by LEM had an average particle size of 2.89 ± 1.02 μm, whereas the SiOC particles obtained by HEM were much finer, with an average size of 0.66 ± 0.2 μm, and had a higher surface area.^62^ This significantly altered the morphology of the anode, providing uniformly distributed active sites, and its small pore size allowed for better connectivity with the current collector. Güneren analyzed the electrochemical impedance spectroscopy (EIS) and concluded that the morphology of the SiOC particles improved after HEM, and the reduction in particle size led to decreased charge transfer resistance, thereby increasing the sodium ion diffusion coefficient. The results showed that the SiOC anode exhibited quite good stability after 200 cycles at 1 A g^−1^. However, carbon black is composed of irregular carbon particles, while carbon nanotubes are tubular structures formed by curling single or multiple layers of graphite sheets. Therefore, the structure of carbon nanotubes enables them to have a higher specific surface area and greater aspect ratio, which is very beneficial for enhancing the anode materials of sodium-ion batteries. Shaikh *et al.* milled red phosphorus with carbon nanotubes (P@CNT) and graphene oxide (P@GO) and evaluated their electrochemical performance as anodes for sodium-ion batteries.^63^ At a low rate (0.1C), the P@CNT and P@GO anodes exhibited excellent capacities, reaching 497 and 458 mA h g^−1^, respectively, at the 200th cycle. At a high rate (3.0C), their capacities were 284 and 211 mA h g^−1^, respectively, further validating their excellent rate performance. In addition to the elemental phosphorus, which can be mixed with carbon-based materials, metal sulfides, oxides, or selenides are often widely used in combination with carbon nanotubes as anode materials for sodium-ion batteries due to their strong redox reactions and low environmental impact. For example, Tammanoon *et al.* combined SnS_2_ with carbon nanotubes (CNT) using a ball milling method to prepare SnS_2_@2%CNT composites, using them as anodes for sodium-ion batteries.^64^ Notably, at 0.1C, this composite demonstrated a high reversible capacity of 548.0 mA h g^−1^. Even after 100 cycles at 1C, it still showed a high reversible capacity of 197 mA h g^−1^. Tammanoon's study found that the simple ball-milled combination of SnS_2_ and CNT generated chemical forces of C–S bonds at approximately 286 eV in the C 1s X-ray photoelectron spectroscopy (XPS) spectrum, resulting in chemical interactions between SnS_2_ and CNT, thereby enhancing the structural stability of the composite material. Furthermore, compared to pure SnS_2_, the binding energies of Sn 3d, S 2p, O 1s, and C 1s in SnS_2_@2%CNT shifted slightly to higher binding energies, further confirming the formation of chemical bonds at the interface of SnS_2_ and CNTs through the ball milling method.

### 3.2 Delamination of sodium-ion battery materials

In the study of anode materials for sodium-ion batteries, layered materials have gained significant attention as one of the promising candidate materials due to their unique crystal structures. Graphite, as a typical layered material, possesses a highly crystalline structure, offering excellent electrical conductivity and structural stability. In lithium-ion batteries, graphite has a theoretical capacity of approximately 372 mA h g^−1^; however, thermodynamic calculations indicate that sodium ions cannot intercalate and deintercalate between the graphite layers in sodium-ion batteries. Recent studies have shown that a series of processing treatments can endow graphite with sodium storage capability. For example, Cao *et al.* reduced the oxygen-containing functional groups on the surface of graphene oxide through a pyrolysis process, thereby removing the oxygen functional groups between the graphite layers and synthesizing expanded graphite.^65^ Expanded graphite can achieve a reversible capacity of 149 mA h g^−1^ at a current rate of 500 mA g^−1^ (2C). Although it exhibits good sodium storage capacity, the synthesis process of this material is overly complicated and energy-intensive. Therefore, Tian *et al.* designed a series of high-defect graphite using a ball-milling method, resulting in an electrochemical sodium storage capacity that is three times greater than that of the original graphite. At a current rate of 0.1 A g^−1^, it can provide a sodium storage capacity of 128 mA h g^−1^. Moreover, at a current rate of 5 A g^−1^, it retains nearly unchanged capacity after 8000 cycles.^66^ It is evident that material delamination through ball milling typically involves separating the active material from conductive agents (such as graphite) or substrates (such as metal foils). This process aims to increase the surface area of the active material or alter its morphology, facilitating contact with the electrolyte and thereby enhancing the energy storage capacity and cycle life of the battery. However, despite various improvements, the specific capacity of graphite remains difficult to exceed 200 mA h g^−1^. The search for higher-capacity anode materials to blend with graphite has become a significant direction in the development of high-capacity sodium-ion battery materials. Lin *et al.* synthesized SeP_2_/C composite materials using a simple plasma-assisted milling (P-milling) method, which involves the delamination of graphite through ball milling.^67^ During the P-milling process, expanded graphite is delaminated and milled to form amorphous carbon. Subsequently, Lin formed SeP_2_ alloy nanoparticles covered with amorphous carbon by combining selenium, red phosphorus, and amorphous carbon. When prepared as the anode for sodium-ion batteries, this structure can mitigate the volumetric expansion of active material particles and promote the formation of Na–Se and Na–P phases, contributing to improved conductivity and enhanced performance of sodium-ion batteries.

Wang and his colleagues synthesized nanoscale FeSb_2_S_4_ anchored on delaminated graphite as an anode for sodium-ion batteries using a planetary ball milling method.^68^ The synthesized FeSb_2_S_4_-G exhibited reversible capacities of 517, 311, and 232 mA h g^−1^ at current rates of 0.2, 1.6, and 4.0 A g^−1^, respectively, which are higher than the reversible capacity of 129 mA h g^−1^ for FeSb_2_S_4_ produced by tube furnace synthesis at 4 A g^−1^. This improvement is attributed to the delamination of graphite during the ball milling process, which weakens the existing chemical bonds, making them easier to break and reform, thereby enhancing the reversibility of the electrode. Additionally, the reduction in particle size of FeSb_2_S_4_ after milling results in an increased number of chemical bonds formed, promoting the formation of chemical bonds between nanocrystalline FeSb_2_S_4_ and delaminated graphite, which positively impacts sodium storage performance. Similarly, Chu from Wang's research group conducted related experiments, wherein nanoscale Sb/Fe_2_S_3_ was obtained by ball milling a mixture of Sb/Fe_2_S_3_ and 15 wt% commercial graphite, anchoring it onto the delaminated graphite composite.^69^ Experimental results indicate that ball milling can delaminate graphite, forming a composite of nanoscale Sb/Fe_2_S_3_ and delaminated graphite. Specifically, during the ball milling process, the mechanical forces acting on the mixture of graphite and Sb/Fe_2_S_3_ may cause the separation or delamination of the graphite layers from Sb/Fe_2_S_3_, resulting in a composite of graphite nanosheets and Sb/Fe_2_S_3_. This leads to the formation of an interface between the graphite nanosheets and Sb/Fe_2_S_3_, which helps to improve the dispersion and electrochemical performance of Sb/Fe_2_S_3_. This delamination effect increases the contact area between Sb/Fe_2_S_3_ and delaminated graphite, promoting interaction between them, thereby enhancing the performance of sodium-ion battery anode materials and providing a simple and effective method for preparing anodes with excellent electrochemical performance. When used as an anode for sodium-ion batteries, the Sb/Fe_2_S_3_-15% electrode prepared by Chu provided reversible capacities of 565, 542, 467, and 236 mA h g^−1^ at current rates of 1, 2, 4, and 10 A g^−1^, respectively, surpassing most Sb-based anodes. Chu also specifically ball-milled commercial graphite, and XRD analysis revealed that the characteristic peaks of graphite nearly disappeared, confirming that ball milling can induce delamination of graphite. Further analysis using XPS demonstrated the formation of chemical bonds between Sb/Fe_2_S_3_ and delaminated graphite during the ball milling process, facilitating the anchoring of nanoscale Sb and nanoscale Sb/Fe_2_S_3_ onto the delaminated graphite.

**Fig. 3:**
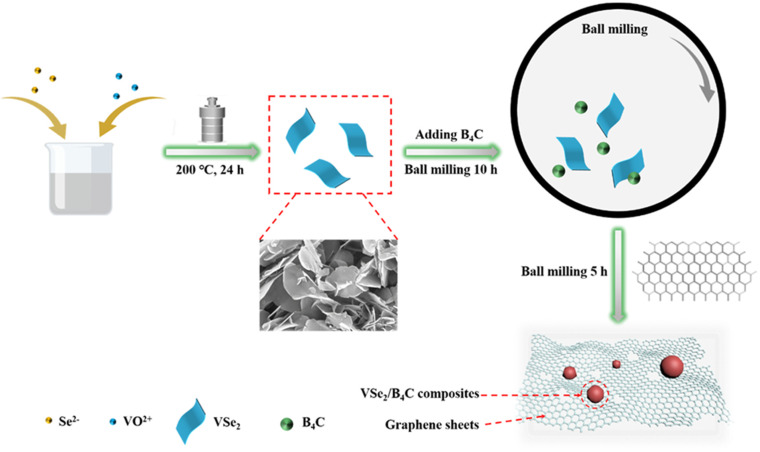
Schematic preparation of the synthesis of VSe_2_/B_4_C@HCG.^70^

As anode materials for sodium-ion batteries, metal selenides generally exhibit faster kinetics and lower discharge plateaus compared to sulfides. On the one hand, metal sulfides have lower conductivity due to the insulating nature of sulfur. On the other hand, selenium, being in the same group as sulfur, has higher electronic conductivity and density than sulfur. As a result, metal selenides possess higher energy density and rate performance than metal sulfides. Zhang *et al.* combined the advantages of VSe_2_ and B_4_C and addressed the drawbacks of VSe_2_ by preparing a VSe_2_/B_4_C@HCG composite using high-energy ball milling, as shown in Fig. 3.^70^ During the preparation process, B_4_C and VSe_2_ nanoparticles were mixed and constrained during the first ball milling stage, effectively mitigating the agglomeration and volume deformation of VSe_2_ during cycling. In the second ball milling stage, VSe_2_/B_4_C was uniformly anchored on highly conductive graphene (HCG) sheets, enhancing the material's stability and conductivity, thereby enabling a higher ion transport rate. When used as an anode for sodium-ion batteries, the VSe_2_/B_4_C@HCG composite delivered a reversible capacity of 407.5 mA h g^−1^ after 450 cycles, with a coulombic efficiency of 98.5%. It exhibited excellent rate performance of 524.2 and 413.7 mA h g^−1^ at different current densities (100 and 500 mA g^−1^, respectively), and after 1000 cycles, it demonstrated ultra-stable long-term cycling performance of 251.6 mA h g^−1^ at 1 A g^−1^. It can be observed that the VSe_2_/B_4_C@HCG composite exhibits excellent electrochemical performance, with high reversible capacity and coulombic efficiency, and superior rate performance at different current densities.

It can be seen that in the preparation of sodium-ion battery anode materials, most of the materials currently exfoliated using ball milling technology are primarily graphite. It is speculated that there are three main reasons: (1) graphite has a layered structure with weak van der Waals forces between the layers, making it relatively easy to exfoliate into graphite nanosheets or flakes during the ball milling process. (2) When graphite is used as the anode in sodium-ion batteries, Na^+^ diffusion during the charging process is hindered. The exfoliated graphite nanosheets produced by ball milling can serve as excellent conductive additives or electrode materials, thereby enhancing the performance of sodium-ion batteries. (3) Graphite, as a traditional material, has a broad application and research foundation in the field of sodium-ion batteries. Therefore, using ball milling to exfoliate graphite can leverage previous research, improving the efficiency and success rate of material preparation. Additionally, red phosphorus also possesses a layered structure. The structure of red phosphorus primarily consists of phosphorus atoms covalently bonded to form layered, chain-like molecules. The layers within this structure are held together by relatively weak van der Waals forces, allowing it to exhibit certain layered characteristics.

Zhang *et al.* used high-energy ball milling (HEBM) to process Ti_3_C_2_T_*x*_ powder and red phosphorus powder at 1000 r min^−1^ for 40 minutes, synthesizing a unique P–O–Ti bonded red phosphorus nanodots/Ti_3_C_2_T_*x*_ (PTCT) composite.^71^ High-energy ball milling effectively layered Ti_3_C_2_T_*x*_ MXenes and amorphous red phosphorus, creating a layered structure that strongly absorbs red phosphorus, offering high reversible capacity and good conductivity, while the layered Ti_3_C_2_T_*x*_ MXene matrix facilitates sodium-ion transfer during cycling. When the PTCT electrode was applied to sodium-ion batteries, the initial capacity of the MXenes was 863.8 mA h g^−1^ at a current density of 50 mA g^−1^, and after 200 cycles, it still achieved 370.2 mA h g^−1^, demonstrating the excellent sodium storage performance of MXene-based materials. It can be seen that ball milling can create new chemical bonds, thereby forming new materials. These chemical bonds enhance the interfacial interaction between substances, promoting electron transfer between them and improving the overall material performance. Additionally, phosphorus can form new chemical bonds not only with transition metal carbides but also with polymers. However, unlike typical layered materials such as graphite, red phosphorus has weaker interlayer interactions and is less prone to slippage, which results in pronounced brittleness on a macroscopic scale.

### 3.3 Synthesis of anode materials for sodium-ion batteries

**Fig. 4:**
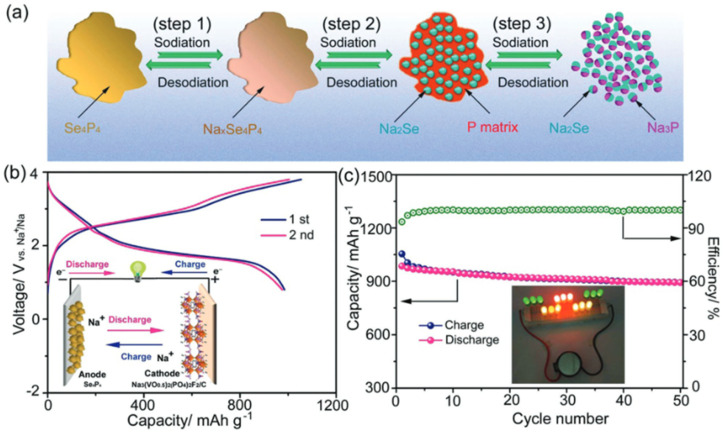
(a) Schematic illustration for the mechanism of Se_4_P_4_ during sodiation/desodiation, (b) charge/discharge curves (inset is the schematicillustration of Se_4_P_4_–Na_3_(VO_0.5_)_2_(PO_4_)_2_F_2_/C full cell), and (c) cycling performance of Se_4_P_4_–Na_3_(VO_0.5_)_2_(PO_4_)_2_F_2_/C full cell (inset is digital picture of afull cell that lights LEDs).^72^

In recent years, researchers have found that the traditional method of simply ball milling carbon materials to alter their microstructure or ball milling them with other materials to form new chemical bonds can improve the performance of anode materials in sodium-ion batteries. However, carbon materials themselves have certain limitations, such as low sodium-ion diffusion rates and limited capacity, which restrict their application to some extent. Therefore, with the emergence of new materials, researchers have begun exploring the use of ball milling technology to synthesize novel materials to improve the electrochemical performance of sodium-ion battery anodes. The advantage of this method lies in its ability to overcome the shortcomings of traditional carbon materials by generating new materials or modifying the microstructure of traditional materials, thereby significantly enhancing the electrochemical performance of anode materials. For example, Lu *et al.* first reported the synthesis of Se_4_P_4_ by ball milling selenium (Se) and phosphorus (P) using a conventional ball milling method, as shown in Fig. 4.^72^ When Se_4_P_4_ was used as the anode for sodium-ion batteries, it exhibited a reversible capacity of 1048 mA h g^−1^ at 50 mA g^−1^, and after 60 cycles, it still retained 804 mA h g^−1^.

However, as the current density increases, its cycling stability and rate performance still need improvement. Moreover, the efficiency of the conventional ball milling method is low, resulting in long milling times and making precise control of the material structure difficult. Therefore, Lin *et al.* mentioned in the previous section used a simple plasma-assisted ball milling method (introducing plasma as an external field during the ball milling process, combining the mechanical energy of ball milling with other physical energies) to mix selenium powder, red phosphorus, and expanded graphite (as mentioned earlier, EG) to synthesize SeP_2_/C composite materials.^67^ Plasma assistance enabled the SeP_2_/C composite to achieve higher efficiency and unique effects in powder refinement, surface modification, and alloying. As an anode for sodium-ion batteries, the SeP_2_/C electrode maintained a reversible capacity of 400 mA h g^−1^ after 500 cycles at 0.5 A g^−1^. Compared to elements like lead (Pb), tin (Sn), and antimony (Sb), non-metal elements such as selenium (Se) and phosphorus (P) undergo significant volume expansion during charge and discharge, leading to structural damage, poor cycling stability, and reactions with the electrolyte. Therefore, Shan *et al.* ball-milled SnO, Sb_2_O_3_, and a certain amount of super P for 15 hours, then added sucrose and ball-milled for another 5 hours to synthesize the SbSn/SP@C composite material.^73^ The prepared SbSn/SP@C electrode achieved 400.3 mA h g^−1^ after 100 cycles, showing good cycling stability and high reversible capacity. Sucrose, as an organic material, is both a renewable resource that is easy to extract and low in preparation cost, while also offering great flexibility and tunability due to its structure and properties being adjustable through chemical synthesis. In the aforementioned experiment, sucrose also acted as a reducing agent, participating in reduction reactions with oxides to produce metals and CO_2_. Therefore, the possible reduction reaction is hypothesized to be:SnO + C_6_H_12_O_6_ → Sn + 6CO_2_ + 6H_2_O2Sb_2_O_3_ + 3C_6_H_12_O_6_ → 4Sb + 18CO_2_ + 18H_2_O

These reactions help to form metal particles, which combine with super P and the carbon coating layer formed during ball milling, resulting in the SbSn/SP@C composite material. Furthermore, the amorphous carbon formed from sucrose carbonization during ball milling not only acts as a reducing agent but also forms a specific core–shell structure to alleviate volume expansion. As a result, the SbSn/SP@C electrode provides an effective carbon-based conductive network, buffering the significant volume changes of the SnSb alloy during cycling, and delivers good electrochemical performance in sodium-ion storage.

In addition to the environmental friendliness and ease of extraction of raw materials, a simple preparation process is also an important factor for large-scale production of sodium-ion battery anode materials. Aydin *et al.* synthesized the SnSe phase in one step using a high-energy ball mill.^74^ The synthesis method in this work is very simple, suitable for large-scale production, solvent-free, and does not require further purification steps or high-temperature treatment. Additionally, no extra conductive carbon was used during electrode preparation, which is different from the aforementioned works, and the synthesized SnSe phase was directly tested as the anode for sodium-ion batteries. SnSe was synthesized by ball milling tin and selenium for 2 hours, achieving a specific capacity of approximately 350 mA h g^−1^ after 100 cycles at 27.5 mA g^−1^. Aydin also performed cyclic voltammetry tests on SnSe, pure metallic Sn and Se, and manually mixed SnSe samples to further observe if there were impurities in the mechanochemically synthesized SnSe composite. It was verified that each sample exhibited different reduction/oxidation peaks, indicating that there was no unreacted Sn or Se in the 2 hour ball-milled sample. It can be seen that even short-time ball milling can yield sodium-ion battery anode materials with good electrochemical performance.

Hu *et al.* proposed a sodium-ion battery anode material with a long cycle life by hybridizing it with a functional conductive polymer.^75^ They prepared a polymer-sulfurized polyacrylonitrile (P-SPAN) mixture from raw phosphorus and polyacrylonitrile (SPAN) in one step using a simple ball-milling method. The ball milling process enabled the formation of P–S bonds between the conductive matrix of this functional conductive polymer and phosphorus, resulting in a robust electrode that could withstand significant volume changes during cycling. Moreover, the C–S–S groups in SPAN serve as ideal carriers for building chemically bonded phosphorus-carbon composite anodes for sodium-ion batteries. During ball milling, the original phosphorus particles, initially irregular and large, became relatively uniform, which facilitated better chemical bonding with SPAN. Hu further clarified the chemical structure of the P-SPAN hybrid using Raman spectroscopy to detect molecular vibration changes. The P-SPAN composite anode achieved high capacities of 1491, 827, and 553 mA h g^−1^ at current densities of 260, 1300, and 3900 mA g^−1^, respectively. Moreover, this hybrid anode exhibited a high coulombic efficiency of over 99% after 100 cycles.

Many researchers use phosphorus mixed with metals or non-metals. On one hand, phosphorus, like carbon, is a high-capacity material that can store large amounts of sodium ions, helping to improve the energy density of batteries. On the other hand, both phosphorus and carbon materials are environmentally friendly, and their production and processing have a lower environmental impact compared to other battery materials, thereby helping to reduce the environmental burden during battery production and use. Literature review shows that phosphorus is often combined with carbon-based materials to prepare sodium-ion battery anode materials, and the possible reasons are as follows: (1) the combination of carbon and phosphorus can further enhance the overall conductivity of the material, facilitating the transport of sodium ions within the electrode material. (2) Phosphorus can stabilize carbon-based materials, helping to prevent volume expansion and structural damage. (3) The combination of carbon-based materials and phosphorus can adjust the surface chemical properties of the material, optimizing the interaction between the electrode and the electrolyte. Fan *et al.* first reported a novel self-healing SnP_3_ material, an anode for sodium-ion batteries.^76^ By using tin (Sn), red phosphorus, and carbon black as raw materials for ball milling, carbon black was chosen because its ductility forms layered sheets along the (0 0 2) crystal plane during ball milling, while the reaction of Sn with P produces brittle SnP_3_ nanoparticles. These brittle nanoparticles are hindered by the ductile components, and through continued ball milling, the tough layers are refined and uniformly wrap around the SnP_3_ nanoparticles. Furthermore, during this process, the carbon in carbon black can tightly bond with SnP_3_ nanoparticles, thereby promoting electron transfer. The SnP_3_/C anode for sodium-ion batteries provides a high capacity of about 810 mA h g^−1^ at 150 mA g^−1^ and maintains the same capacity over 150 cycles, which is excellent compared to P, Sn, and Sn–P composite anodes for sodium-ion batteries. Fan, through scanning electron microscope (SEM), transmission electron microscope (TEM), and XPS measurements, found that strong bonding interactions formed between Sn and P after ball milling. This highly reversible conversion reaction successfully mitigated the accumulation of pulverization and agglomeration, revealing the self-healing structure of the SnP_3_/C composite material and enhancing the cycle stability of the anode. This shows that carbon black, as a low-cost and stable material, is highly suitable for use as a conductive agent or filler in synthesizing sodium-ion battery anode materials.

Sun and colleagues mixed V_2_O_5_, super P, and carbon nanotubes using ball milling for two days, then introduced carbon disulfide for curing and adjusted the annealing temperature to obtain three different crystal types: V_5_S_8_, V_3_S_4_, and V_3_S_5_.^77^ Comparisons revealed that super P and carbon nanotubes can tightly connect with V_5_S_8_ nanosheets, and this conductive structure facilitates the transport of ions or electrons. Additionally, the dark-field images and corresponding elemental mapping of V_5_S_8_ further confirmed the uniform distribution of V and S elements after ball milling. As an anode for sodium-ion batteries, V_5_S_8_ outperforms V_3_S_4_ and V_3_S_5_ in enhancing battery performance, providing a specific capacity of 918 mA h g^−1^ at a current density of 0.1 A g^−1^ (for 100 cycles). Sun also fabricated a full sodium-ion battery using a V_5_S_8_ anode and Na_3_V_2_(PO_4_)_2_O_2_F cathode, showing a high energy density of 165 W h kg^−1^. Furthermore, Li *et al.* first reported the successful preparation of Na_2.60_Fe_1.70_(SO_4_)_3_@GO composites *via* ball milling. This composite embeds uniform Na_2.60_Fe_1.70_(SO_4_)_3_ particles into a crosslinked graphene oxide (GO) network structure.^78^ Li fabricated an anode for sodium-ion batteries, demonstrating excellent rate performance, and designed a symmetric battery with Na_2.60_Fe_1.70_(SO_4_)_3_@GO as both the cathode and anode, reaching 60 mA h g^−1^ at 0.2C. Raman spectroscopy analysis revealed that peaks below 1000 cm^−1^ correspond to the Raman vibrational motion of SO_4_^2−^, and the composite material shows a weak –OH peak, indicating that the carbon protective layer formed in the crosslinked graphene oxide structure *via* ball milling effectively reduces the impact of water molecules on the active material. Kang *et al.* used sodium fluoride (NaF) and titanium fluoride (TiF_3_) as precursors (in a molar ratio of 5 : 3), sealed them in a silicon nitride bottle, and mixed them using high-energy ball milling at 400 rpm for 12 hours, thereby synthesizing Na_5_Ti_3_F_14_ cryolite powder.^79^ After a series of treatments, the active material Na_5_Ti_3_F_14_ was mixed with conductive carbon (super P, carbon black, and multi-walled carbon nanotubes) at 300 rpm for 12 hours, and high-energy ball milling was performed again to prepare NTF/C nanocomposites. Kang used TEM and energy-dispersive spectroscopy (EDS) to analyze the particle size and atomic distribution of NTF/C nanocomposites. The Na, Ti, F, and C elements were uniformly distributed in approximately 600 nm NTF/C particles, indicating that the carbon coating of ball-milled NTF/C particles performed well. During charge–discharge cycles at a current density of 10 mA g^−1^, Na_5_Ti_3_F_14_ provided a specific capacity of approximately 425 mA h g^−1^. Despite the presence of conversion reactions, a high coulombic efficiency of over 99% was achieved at 1 A g^−1^. It can be seen that in composite materials, graphene oxide provides a stable support structure, helping to uniformly embed the active material (Na_2.60_Fe_1.70_(SO_4_)_3_) into its network structure. This uniform dispersion helps improve the utilization of the active material and increases the battery's capacity and cycling stability. Based on the above, although different materials are used, notable components associated with phosphorus include graphene oxide, carbon black, and carbon nanotubes, all of which are carbon-based materials. Therefore, carbon-based materials play a vital role in the anodes of sodium-ion batteries, improving battery performance and cycling stability. Moreover, different carbon-based materials have varying roles and effects in constructing conductive structures and enhancing electrode performance. Therefore, when designing sodium-ion battery anodes, selecting the appropriate carbon-based material, along with the ratio and structural design of other active materials, will have a significant impact on battery performance.

In carbon-based materials, carbon nanotubes are often improved using ball milling technology, allowing them to serve as the primary active material in the synthesis of sodium-ion battery anode materials. Upon reflection, we believe that one reason is that during the ball milling process, carbon nanotubes may undergo mechanical shearing and collisions, leading to more uniform dispersion. This helps prevent the aggregation or clumping of carbon nanotubes in the material, thus enhancing their dispersion and uniformity. Another reason is that during the ball milling process, friction and collisions may occur between carbon nanotubes and other materials, promoting mixing and interactions. This facilitates the effective integration of carbon nanotubes with other functional materials, further improving the performance of the composite material. Therefore, optimizing the structure of carbon nanotubes during the synthesis of sodium-ion battery anode materials to enhance the properties of newly synthesized materials is highly beneficial. For example, Zhao and colleagues successfully prepared PbSe nanoparticles and carbon nanotube (PbSe@CNTs) composites through simple mechanical ball milling.^80^ When PbSe@CNTs was used as the anode for sodium-ion batteries, it exhibited a high reversible capacity of 597 mA h g^−1^ at 20 mA g^−1^. Zhao utilized lead recovered from waste lead-acid batteries and commercial selenium powder, ball-milled with carbon nanotubes (CNTs), and found that ball milling caused the reaction between Pb and Se powders to form a new material, PbSe. The CNTs were uniformly connected to PbSe nanoparticles, effectively increasing their electronic conductivity, reducing volume expansion, and enhancing the electrochemical performance of the sodium-ion battery. Ihsan and colleagues first used a planetary ball mill to prepare Sb_2_Te_3_ powder by ball milling antimony (Sb) and tellurium (Te), and then further ball-milled it with functionalized carbon nanotubes (CNTs) in a 90 : 10 weight ratio to prepare the Sb_2_Te_3_/CNT_10_ composite.^81^ CNTs, serving as a conductive framework, can buffer the volume changes of Sb_2_Te_3_ particles during the sodium electrochemical reaction. During the charge and discharge cycles of the sodium battery, Sb_2_Te_3_ particles undergo volume changes, and CNTs can act as a flexible support structure to alleviate the impact of these changes on the electrode, improving its structural stability. The numerous defects introduced during the ball milling process provide pathways for rapid ion transport. These defects enhance the conductivity of the composite material and promote the rapid transport of sodium ions within the electrode material, improving the kinetic response and leading to the excellent performance of the Sb_2_Te_3_/CNT_10_ composite as a sodium-ion battery anode.

### 3.4 Doped materials for sodium-ion batteries

For sodium-ion batteries, doping is also a commonly used step in the preparation of anode materials, similar to grinding, mixing, exfoliating, and synthesizing, utilizing ball milling technology. Doping involves introducing other substances or elements into the anode material to alter its electrochemical properties or structural characteristics, thereby optimizing battery performance. The application of ball milling technology in the doping process primarily involves thoroughly mixing the dopants with the substrate material to achieve uniform dispersion and promote chemical reactions or interactions between the dopants and substrate material. This ball milling process can enhance the uniform distribution of dopants and the contact area with the substrate material, thereby improving the doping effect and enhancing the performance of the battery. Therefore, doping is one of the important steps in the preparation of sodium-ion battery anode materials, and ball milling technology provides an effective means to achieve doping. Doping of battery materials can be achieved through various methods, including but not limited to.

**Fig. 5:**
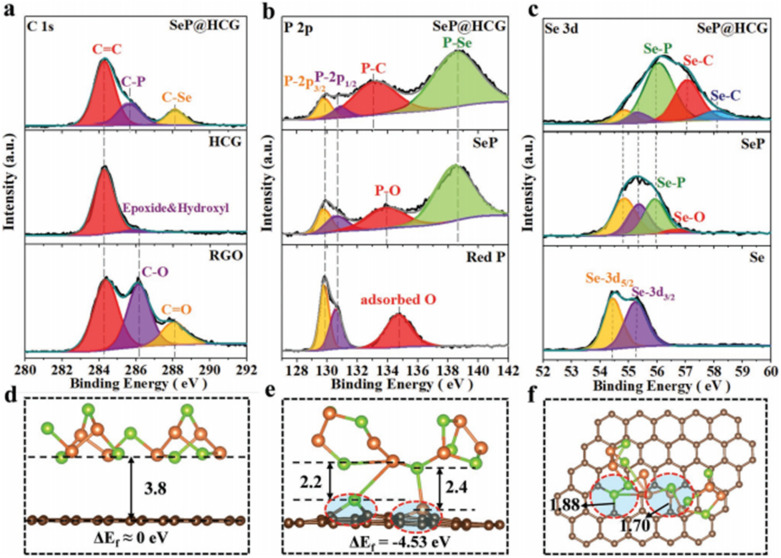
XPS characterization: (a) C 1s spectrum of HCG, RGO, and SeP@HCG, (b) P 2p spectrum and (c) Se 3d spectrum. And optimized configurations for the adsorption of SeP on various graphene substrates (green sphere for Se, light brown sphere for P, small dark brown sphere for C), (d) side view of perfect graphene (C: Se: P = 60: 8: 8), (e) side view of graphene with two “C” defects (C: Se: P = 58: 8: 8), and (f) planform of graphene with two “C” defects.^84^

Firstly, elemental doping refers to the introduction of other elements into the anode material of sodium-ion batteries to alter its structure and electrochemical properties. For example, Kim *et al.* utilized high-energy mechanical ball milling to incorporate sulfur (S) into nickel (Ni) and phosphorus (P), forming a negative ion exchange type NiP_2−*x*_S_*x*_ solid solution, which was then tested for its electrochemical performance as an anode for sodium-ion batteries.^82^ At a current density of 500 mA g^−1^, it delivered a reversible capacity of 299 mA h g^−1^ after 200 cycles. During the ball milling process, S gradually replaced a portion of P, forming the NiP_2−*x*_S_*x*_ (*x* = 0, 0.5, 1.0, 1.5, and 2.0) solid solution. Kim discovered through XRD testing that the diffraction pattern of NiP_2−*x*_S_*x*_ is similar to that of NiP_2_ and NiS_2_, without any obvious phase separation observed. As the sulfur substitution increased, the XRD peaks gradually shifted to lower 2*θ* values, indicating the presence of a complete substitution solid solution between NiP_2_ and NiS_2_. Further confirmation from the high-resolution powder diffraction (HRPD) data obtained using synchrotron X-ray beams validated that the ball-milled NiP_1.5_S_0.5_ is a solid solution between NiP_2_ and NiS_2_. Scanning transmission electron microscopy (STEM) images and EDS elemental mapping images showed that Ni, P, and S elements are uniformly distributed in the NiP_1.5_S_0.5_ nanoparticles. This solid solution affects the redox reactions of the end members and activates the reaction between P and Na ions, thereby increasing the discharge capacity. In addition, Lu *et al.* proposed the preparation of high-performance dual-atom-doped carbon (C) materials through ball milling using low-cost corn starch for pretreatment and thiourea (CH_4_N_2_S) as a precursor.^83^ Corn starch was first pretreated as a carbon source, resulting in the formation of oxygen-containing functional groups (C–OOH) in the carbon-based material, which facilitates the easier doping of nitrogen and sulfur elements into the carbon matrix. After ball milling with CH_4_N_2_S as the nitrogen and sulfur source, the incorporation of sulfur effectively expanded the interlayer spacing, while the incorporation of nitrogen promoted the formation of more defects and active sites, making the carbon material more disordered and providing more pathways for sodium ion transport. The sodium-ion battery anode prepared from (N, S)–C exhibited a reversible capacity approaching 400 mA h g^−1^ after 200 cycles at a current density of 500 mA g^−1^. Furthermore, Sang and colleagues ball-milled amorphous SeP with highly conductive crystalline graphene (HCG) to prepare SeP@HCG composite materials, as shown in Fig. 5.^84^ The doping process primarily involves mixing SeP with HCG material to prepare the composite. Amorphous SeP and HCG materials are mixed in a specific ratio and then subjected to ball milling. During the ball milling process, SeP is fully combined with the carbon-based material, and the mechanical alloying effect leads to the replacement of a portion of the lattice carbon in HCG by selenium (Se) and phosphorus (P), resulting in a doped structure. By fabricating the SeP@HCG composite material into a sodium-ion battery anode, Sang achieved long-term cycling stability with bare sodium as the electrode for the first time, maintaining a stable capacity of 732 mA h g^−1^ over 500 cycles at a current density of 0.65 A g^−1^. It is evident that this structure allows the interconnected graphene network framework to create a free space, effectively mitigating the significant volume changes during the redox reactions of SeP, which is crucial for ensuring high cycling stability. Additionally, the graphene network structure throughout the composite material serves as a pathway for the rapid transport of sodium ions and other electrons, accelerating the redox process in the SeP@HCG electrode and thus providing excellent rate performance.

Secondly, ion doping refers to the introduction of additional ions into the negative electrode materials of sodium-ion batteries to alter their conductivity or cycling stability. For example, Gu *et al.* doped Sn^4+^ into NaTi_2_(PO_4_)_3_ using a ball mill, followed by high-temperature calcination to produce NaSn_0.02_Ti_1.98_(PO_4_)_3_/C composite material.^85^ When prepared as an anode for sodium-ion batteries, it was found that the initial discharge capacity was 108 mA h g^−1^ under 1C conditions. Moreover, under 10C conditions, after 1400 cycles, the capacity retention rate was 84.2%. The doping of Sn^4+^ increased the lattice spacing of NaTi_2_(PO_4_)_3_, which broadened the transport pathway for sodium ions within the lattice structure, thereby accelerating the diffusion coefficient of sodium ions. It can also be observed from the NaSn_0.02_Ti_1.98_(PO_4_)_3_/C composite material that carbon elements were also doped, aimed at effectively reducing the charge transfer impedance and further enhancing the ion diffusion coefficient. Additionally, Gu demonstrated that the distribution of tin (Sn) and carbon (C) in the EDS mapping was consistent with that of oxygen (O), titanium (Ti), phosphorus (P), and sodium (Na), indicating that ball milling resulted in the uniform distribution and successful doping of Sn and C in the NaSn_0.02_Ti_1.98_(PO_4_)_3_/C composite material. Subsequently, EIS results confirmed that the carbon coating reduced the charge transfer impedance of NaTi_2_(PO_4_)_3_ and increased the diffusion coefficient of sodium ions. Therefore, NaSn_0.02_Ti_1.98_(PO_4_)_3_/C exhibits good electrochemical performance and rapid charge–discharge capabilities.

Functional doping refers to the incorporation of compounds or functional groups with specific functions to enhance the conductivity or electrochemical reactivity of sodium-ion battery anode materials. For example, Ihsan *et al.*, previously mentioned in the last two sections, synthesized Sb_2_Te_3_ powder by ball milling Sb and Te, and subsequently further milled the obtained Sb_2_Te_3_ powder with functionalized carbon nanotubes (CNTs) to prepare Sb_2_Te_3_/CNT_10_ composite material.^81^ XPS, TEM, and first-principle calculations demonstrated that during the ball milling process, chemical bonds such as Sb–C, Te–C, Sb–O–C, and Te–O–C were formed between Sb, Te, and the functional groups of the carbon nanotubes. The formation of these chemical bonds resulted in interactions between the carbon atoms in the carbon nanotubes and the Sb and Te atoms in Sb_2_Te_3_, allowing the functionalized carbon nanotubes to act as a conductive framework for Sb_2_Te_3_ particles. This framework can buffer the volume changes that occur during electrochemical reactions with sodium, thereby enhancing the structural stability of the composite material and improving its electrochemical performance. When tested for electrochemical performance, after being prepared as a sodium-ion battery anode, it maintained a capacity retention rate of over 97.5% after 300 cycles at a current density of 100 mA h g^−1^, with an energy density of approximately 229 W h kg^−1^ at 0.5 °C. In addition, Zhou *et al.* prepared the Sb-graphite-NaF (SCF) ternary composite material using ball milling.^86^ After ball milling, NaF was uniformly dispersed in the material and acted as a functional dopant, promoting the accumulation of fluorine-containing components on the Sb anode surface, forming a stable solid electrolyte interphase (SEI) film rich in NaF, primarily consisting of inorganic components, which helped stabilize the electrode/electrolyte interface. The stable SEI film works synergistically with graphite to suppress the volume expansion of Sb particles during sodium storage, while also improving conductivity and accelerating Na^+^ diffusion at both room and low temperatures. When prepared as a sodium-ion battery anode, experimental results showed that in a propylene carbonate-based electrolyte, the SCF electrode exhibited a discharge capacity of 458.4 mA h g^−1^ after 160 cycles at 500 mA g^−1^ at 20 °C. When paired with a diethyl carbonate-based electrolyte, the SCF electrode maintained a specific capacity of 299.8 mA h g^−1^ after 120 cycles at −20 °C, with a capacity retention rate of 86.2%.

Compared to other traditional doping techniques, ball milling doping exhibits a range of unique advantages. Firstly, ball milling can achieve uniform dispersion of dopants in a short period of time. The intense mechanical forces generated during the ball milling process enable dopant elements to be evenly distributed within the matrix material, thus avoiding the non-uniform doping issues associated with traditional methods, such as solution-based techniques. Furthermore, the mechanical interactions between dopant elements and the matrix material during milling help to promote chemical reactions or interactions, thereby enhancing the structural stability of the composite material and improving its electrochemical performance. More importantly, the ball milling doping method requires only grinding and simple post-processing during the preparation of electrode materials, eliminating the need for additional steps such as etching, transferring, or reduction, thus avoiding the high costs and complex equipment required in methods like CVD and thermal treatment. In comparison with these methods, ball milling doping is not only simple to operate, cost-effective, and environmentally friendly, but it also does not rely on complex solvents or expensive equipment, making it particularly suitable for large-scale, efficient production. As a result, it shows significant advantages in industrial applications.

## 4. Conclusions and outlook

In the process of preparing sodium-ion battery anode materials, ball milling technology has gradually taken on an important role among various techniques due to its simplicity, efficiency, and ability to alter the particle size and morphology of materials. This paper discusses the preparation of anode materials based on different principles of interactions between substances during ball milling, including ball milling crushing/mixing principles, ball milling delamination principles, ball milling synthesis principles, and ball milling doping principles. These ball milling principles effectively activate the material's surface, increasing active sites and reaction activity, thus enhancing the electrochemical performance of the resulting sodium-ion battery anodes. The advantages and disadvantages of preparing sodium-ion battery anode materials using the ball milling method are summarized below.

(1) The simplest application of ball milling is to crush substances, which can alter the crystal structure of materials, thereby enhancing the cycling stability and electrochemical performance of sodium-ion batteries. However, most of the materials that can be directly prepared into sodium-ion battery anodes after ball milling are carbon-based materials, such as hard carbon, carbon nanotubes, and carbon black, which are commonly used anode materials but have limited interaction with sodium ions. Therefore, the mixing of carbon-based materials has attracted attention, as it allows for the combination of various advantages of different substances. Additionally, phosphorus, sulfides, selenides, and oxides can also interact with carbon-based materials through ball milling; this mixing can fully utilize the conductivity and structural stability of the carbon-based materials. On the other hand, when combined with the strong redox reactivity of these materials, it can effectively enhance the performance of sodium-ion batteries.

(2) Similar to crushing, ball milling also employs a technique that alters the crystal structure of materials without synthesizing new substances, known as delamination. The materials typically subjected to delamination *via* ball milling are layered materials, which have attracted considerable attention in sodium-ion batteries due to their unique crystal structures. Graphite, as a typical layered material, can significantly enhance its sodium storage performance through ball milling delamination techniques. After ball milling, the resulting graphite nanosheets possess a larger surface area and more active sites, contributing to improved electrochemical performance in sodium-ion batteries. Moreover, graphite can also be compounded with metal sulfides, selenides, and other materials, further enhancing conductivity and cycle life. This makes graphite and its composite materials strong candidates for sodium-ion battery anodes.

(3) Ball milling can also induce reactions between two substances, leading to the formation of new materials, mostly involving phosphorus or carbon-based materials mixed with metals, nonmetals, oxides, sulfides, fluorides, and polymers to prepare composites. However, the reactions between fluorides, sulfides, and carbon elements often produce toxic substances, which can lead to environmental pollution. Therefore, there is a need to use safer and more environmentally friendly materials to replace the aforementioned substances.

(4) The categories of ball milling doping also represent a significant direction, including elemental doping, ionic doping, and functional doping. By replacing one element with another, incorporating an ion into another substance, or introducing a functional substance into another material, we can understand the changes in chemical bonds and their effects on battery performance. However, the influence of these on sodium storage mechanisms, potential side reactions during ball milling doping, and the selection of suitable dopants are all issues that need to be addressed. Currently, theoretical calculations are increasingly becoming a necessary step before conducting experiments; density functional theory can be used to investigate the structure, stability, and electronic properties of sodium storage materials. Utilizing computational chemistry methods, one can simulate the potential chemical reactions and side reactions that may occur during ball milling doping, predicting and understanding reaction pathways and products under different conditions, thereby guiding experimental design and optimizing the ball milling doping process. Moreover, by calculating the electronic structure, band structure, and interatomic interactions of materials through computational chemistry, one can evaluate the effects of different dopants on material performance and select the most suitable dopants. Additionally, by calculating the doping energy and stability of materials, one can predict the doping locations and concentrations of dopants to achieve precise doping.

(5) There is still room for improvement in the parameters of ball milling (such as milling time, speed, media, materials, and temperature/environment); for example, introducing plasma during ball milling can harness the energy generated by plasma in conjunction with mechanical energy to alter the physical properties and chemical reaction processes of materials. This method allows for precise control over material properties by regulating plasma parameters (such as temperature, density, composition, *etc.*) and ball milling parameters (such as milling time, speed, and media) to obtain materials with specific structures and properties.

Despite some shortcomings in the ball milling of sodium-ion battery anode materials, processes such as crushing, mixing, delamination, synthesis, and doping have shown significant development potential. With the advancement of materials science, the application prospects of ball milling are becoming increasingly broad. Below are the prospects for the future development of ball milling technology.

(1) The flexibility and efficiency of ball milling technology give it great potential in the synthesis of multifunctional materials. In the future, ball milling can serve as an effective method for developing composite materials with excellent electrochemical properties. For example, new catalysts can be synthesized *via* ball milling, which can further optimize the microstructure, cross-sectional characteristics, and particle size distribution of the catalyst, leading to higher activity or selectivity and thus significantly enhancing its catalytic performance. Furthermore, composites prepared by ball milling not only perform well in traditional catalytic reactions but also achieve synergistic effects in more complex energy conversion and storage processes. For instance, in water electrolysis and carbon dioxide reduction reactions, multicomponent catalytic materials prepared by ball milling can enhance reaction efficiency and reduce energy consumption by optimizing the interactions between substances. At the same time, these composite materials may also possess a high specific surface area and excellent electronic/ionic conductivity, thereby playing a more important role in batteries, capacitors, and other energy storage devices.

(2) By integrating advanced computational chemistry and materials design methods, future research can further optimize ball milling process parameters (such as milling time, speed, and media) to obtain higher performance anode materials. Specifically, first-principles calculations (such as density functional theory, DFT) can accurately describe the electronic structure and phase transition behavior of materials at the atomic level. Through DFT calculations, researchers can predict the structural changes, energy variations, and reaction pathways that materials may undergo during the ball milling process. This is particularly useful in the study of composite or doped materials, where first-principles can help predict the impact of dopant elements on material performance. For example, calculating the solubility, diffusion coefficients, and potential interfacial interactions of dopants during ball milling provides more detailed guidance for optimizing the milling process. Molecular dynamics (MD) simulation is an effective tool that can predict the microscopic changes in materials under different ball milling parameters by simulating the impact, friction, and compression forces exerted on particles during milling. MD simulations can model the movement and interactions of material particles under various milling times, speeds, and ball-to-material ratios, providing deep insights into the effects of these parameters on particle size, morphology, structural stability, and performance. This simulation not only aids in optimizing experimental design but also provides theoretical support for practical operations. In addition, machine learning and data mining techniques, which are among the most advanced computational methods today, can analyze large amounts of experimental data and identify potential correlations between ball milling parameters and material performance. By constructing predictive models, machine learning can help researchers extract patterns from complex experimental data and enable intelligent optimization of the ball milling process. For example, using regression analysis or neural network algorithms, machine learning can predict the electrochemical performance, structural stability, and other indicators of materials under different conditions. This not only accelerates experimental design but also helps identify the optimal parameter combinations across numerous experimental conditions, reducing the time and cost of repetitive experiments.

(3) In addition to its widespread use in the preparation of sodium-ion battery anode materials, the flexibility and efficiency of ball milling technology also offer significant prospects for the synthesis of anode materials in magnesium-ion, zinc-ion, and potassium-ion batteries. As research on these new battery technologies deepens, the versatility of ball milling can provide suitable anode materials for different ion batteries, contributing to the development of more efficient and safer energy storage systems. Moreover, ball milling technology is not limited to the preparation of anode materials but can also extend to the synthesis of cathode materials. By subjecting both anode and cathode materials to ball milling, better compatibility and synergy can be achieved, enhancing the overall performance of the battery. This comprehensive material optimization will promote further improvements in the energy density and cycle life of sodium-ion batteries.

As ball milling technology continues to improve and research on anode materials deepens, the study of anode materials in the field of sodium-ion batteries will continue to flourish. This review aims to provide valuable insights for exploring scalable and controllable preparation methods as well as improving the electrochemical performance of electrodes. We also hope it will inspire more promising applications of ball milling technology in the preparation of sodium-ion battery anode materials.

## Data availability

No data was used for the research described in the article.

## Author contributions

LW: conceptualization, data curation, formal analysis, investigation, writing – original draft. HD: supervision, visualization, writing – review & editing. TB: supervision, visualization, writing – review & editing. YH: writing – review & editing.

## Conflicts of interest

The data that support the findings of this study are available from the corresponding author upon reasonable request.
